# Supplementary material for: A Likelihood Approach for Real-Time Calibration of Stochastic Compartmental Epidemic Models
Source: PLoS Comput Biol. 2017 Jan 17;13(1):e1005257. doi: 10.1371/journal.pcbi.1005257 (PMC5240920; doi:10.1371/journal.pcbi.1005257)
Supplement: S1 File — (TAR.GZ) [file pcbi.1005257.s014.tar.gz › HSPH_Online-SI-Revision/EnKF07/n10-severe/n10-severe_EnKF_table0.pdf]

1	R0	{1.68952, 11.8402}	{2.48939, 5.37361}	{{1.10804, 0.139886}, {3., 16.4166}}	{61.601, 79.281}	inf	1046.	550.915	{23.1849, 786.362}	89.4487	0.752738	newly	589.	1629.63	{36., 7622.}	268.817	0.675749	Reff	0.898487	0.69484	{0.195312, 1.71052}	57.915	ar	6872.	17797.	{15555., 24033.}	158.978
2	R0	{1.71739, 9.62661}	{2.51245, 9.85344}	{{1.76842, 3.61709}, {3., 15.9292}}	{46.2945, 54.8279}	inf	945.	351.247	{124.376, 499.545}	65.793	0.54035	newly	711.	275.33	{76., 506.}	65.7314	0.575655	Reff	0.984408	0.552803	{0.302319, 0.98302}	51.7155	ar	6874.	16636.2	{15764., 17835.}	142.016
3	R0	{1.48936, 6.23975}	{2.35369, 6.70258}	{{0.585653, 0.486825}, {3., 10.6805}}	{85.9214, 79.243}	inf	625.	614.137	{41.4662, 1146.07}	125.509	0.665732	newly	640.	841.96	{30., 1881.}	147.547	0.748786	Reff	0.888701	0.602334	{0.103145, 1.05413}	49.8635	ar	5666.	17432.3	{15564., 20119.}	207.665
4	R0	{1.62394, 7.51135}	{2.63566, 5.56719}	{{1.90878, 3.5451}, {3., 7.24679}}	{63.9719, 25.883}	inf	753.	475.521	{63.2814, 1217.88}	92.4192	0.635848	newly	670.	531.43	{41., 1640.}	100.825	0.639476	Reff	0.945133	0.563441	{0.0981264, 1.52078}	66.1927	ar	6417.	17514.8	{15333., 24764.}	172.943
5	R0	{1.53632, 8.57652}	{2.67409, 2.62993}	{{1.92114, 1.94454}, {3., 3.46217}}	{75.3873, 69.3357}	inf	707.	634.271	{126.677, 1428.09}	97.1041	0.490871	newly	572.	1555.45	{122., 5645.}	235.561	0.444303	Reff	0.929627	0.676761	{0.0461347, 1.92095}	66.2832	ar	6260.	17378.	{15011., 25355.}	177.604
6	R0	{1.47166, 5.7434}	{2.61499, 3.61724}	{{1.52211, 1.02088}, {3., 5.283}}	{84.7219, 37.0191}	inf	531.	603.053	{22.7117, 496.168}	156.33	0.844647	newly	615.	708.84	{32., 3040.}	147.324	0.778554	Reff	0.960847	0.619567	{0.234728, 0.790568}	54.9374	ar	5650.	17320.5	{15176., 23641.}	206.557
7	R0	{1.48179, 8.87369}	{2.65959, 5.88009}	{{1.92596, 3.52046}, {3., 8.07783}}	{79.4853, 33.7357}	inf	480.	386.545	{98.8396, 286.889}	102.993	0.529984	newly	360.	436.66	{70., 1141.}	121.172	0.471834	Reff	0.902706	0.661054	{0.0797921, 1.92139}	65.6563	ar	5233.	17255.8	{15014., 23863.}	229.749
8	R0	{1.53669, 6.47807}	{2.73206, 12.5785}	{{2.18471, 6.75574}, {3., 16.5032}}	{77.7888, 94.1702}	inf	675.	412.116	{53.5722, 361.049}	88.789	0.554097	newly	686.	222.32	{21., 212.}	89.1195	0.822081	Reff	0.902344	0.684047	{0.220576, 1.30257}	49.3102	ar	5934.	17031.5	{15491., 18207.}	187.015
9	R0	{1.57227, 6.20989}	{2.28824, 9.13253}	{{0.306265, 5.43734}, {3., 13.6301}}	{78.6956, 60.5001}	inf	741.	304.502	{28.5415, 483.967}	75.6968	0.696732	newly	787.	212.69	{22., 291.}	83.3177	0.887004	Reff	1.05279	0.457915	{0.0685096, 0.6706}	56.6035	ar	6136.	17164.9	{16418., 17381.}	179.742
10	R0	{1.54936, 6.50938}	{2.19855, 8.67567}	{{0.277802, 5.24945}, {3., 13.7312}}	{79.6101, 56.1191}	inf	717.	312.129	{15.8486, 471.217}	75.6577	0.705335	newly	740.	234.01	{15., 311.}	82.2149	0.865477	Reff	0.879107	0.462788	{0.0585076, 0.679742}	49.4847	ar	6275.	17584.4	{16673., 17790.}	180.23
11	R0	{1.42179, 3.18601}	{2.50482, 7.99383}	{{2.03936, 1.12591}, {3., 18.8147}}	{90.6455, 224.962}	inf	449.	424.801	{56.3619, 606.635}	80.1697	0.395529	newly	893.	1085.56	{32., 1903.}	146.39	0.766842	Reff	0.921746	0.570351	{0.0548097, 1.4428}	61.2748	ar	5322.	17801.8	{16255., 19975.}	234.495
12	R0	{1.48035, 6.46183}	{2.44683, 8.35808}	{{0.455929, 1.23535}, {3., 18.7838}}	{85.1708, 122.928}	inf	718.	526.228	{34.4363, 1452.91}	94.9241	0.615824	newly	750.	1160.98	{19., 2407.}	176.597	0.77965	Reff	0.99065	0.601967	{0.0619138, 1.39009}	60.2454	ar	5793.	18343.5	{16677., 21189.}	216.65
13	R0	{1.75755, 8.53854}	{2.70986, 12.6241}	{{2.24113, 6.87992}, {3., 17.5628}}	{54.1838, 65.7197}	inf	1074.	493.802	{61.3624, 732.139}	89.3145	0.705819	newly	871.	257.7	{27., 256.}	86.6935	0.868561	Reff	0.980361	0.672756	{0.137469, 1.17974}	54.094	ar	6832.	17383.6	{15375., 21729.}	154.443
14	R0	{1.47355, 4.89451}	{2.5871, 6.40112}	{{1.5799, 3.62249}, {3., 8.67455}}	{75.5695, 54.1715}	inf	528.	505.467	{240.806, 533.836}	83.7366	0.32493	newly	648.	665.3	{141., 781.}	110.972	0.471599	Reff	0.862321	0.601911	{0.0960513, 0.974427}	54.2869	ar	5710.	18172.4	{15965., 24444.}	218.256
15	R0	{1.62071, 5.98665}	{2.23006, 5.31253}	{{1.68564, 3.99576}, {3., 4.37796}}	{47.1995, 50.797}	inf	906.	883.266	{63.3539, 2454.05}	106.56	0.617621	newly	967.	1337.43	{62., 5626.}	154.849	0.693262	Reff	0.893659	0.552608	{0.169176, 1.68035}	60.3191	ar	6635.	19250.	{16442., 26826.}	190.206
16	R0	{1.67268, 7.7256}	{2.52971, 6.30892}	{{1.48791, 3.58032}, {3., 8.54142}}	{57.5532, 29.9534}	inf	927.	433.569	{205.261, 649.536}	78.101	0.538392	newly	767.	561.58	{136., 777.}	95.4055	0.539466	Reff	0.766087	0.540028	{0.0831028, 0.762245}	51.6895	ar	6947.	17635.5	{15655., 23646.}	153.858
17	R0	{1.67805, 11.1417}	{2.5804, 6.57985}	{{1.642, 3.59634}, {3., 8.56885}}	{54.8477, 40.944}	inf	929.	427.852	{159.576, 437.823}	85.5273	0.614993	newly	581.	567.91	{121., 549.}	110.105	0.489825	Reff	1.07479	0.670959	{0.194452, 0.955582}	56.4502	ar	6625.	18269.2	{16337., 25030.}	175.761
18	R0	{1.66743, 9.99046}	{2.79507, 11.5544}	{{2.28027, 6.62437}, {3., 14.7359}}	{67.6276, 41.935}	inf	904.	364.894	{90.634, 327.359}	87.4576	0.723272	newly	639.	213.84	{40., 219.}	87.6307	0.818806	Reff	0.998457	0.645954	{0.189393, 1.12698}	48.5556	ar	6899.	16877.	{15591., 17275.}	144.63
19	R0	{1.70787, 9.85478}	{2.63703, 2.65526}	{{1.90541, 1.88386}, {3., 3.50518}}	{54.4047, 73.0561}	inf	940.	710.838	{197.634, 1409.08}	79.5815	0.469755	newly	617.	1816.23	{190., 6859.}	243.272	0.407879	Reff	0.877333	0.684934	{0.123451, 1.91907}	71.5914	ar	6867.	17794.1	{15217., 25338.}	159.124
20	R0	{1.53809, 10.5848}	{2.59497, 4.91979}	{{1.9515, 3.4132}, {3., 5.96674}}	{76.5896, 53.5202}	inf	660.	345.47	{88.5959, 521.497}	90.7506	0.679675	newly	432.	719.53	{64., 818.}	179.887	0.618873	Reff	1.05928	0.518516	{0.0897256, 1.06403}	68.8371	ar	6108.	16385.2	{14515., 20123.}	168.258
21	R0	{1.59925, 6.74479}	{2.25258, 8.90602}	{{0.2777, 5.28708}, {3., 13.6258}}	{76.9469, 56.2488}	inf	837.	317.707	{22.0545, 511.117}	79.4709	0.805301	newly	789.	226.42	{10., 309.}	84.6058	0.942018	Reff	0.912212	0.464628	{0.0601399, 0.699677}	50.6987	ar	6411.	17563.8	{16749., 17924.}	173.964
22	R0	{1.38779, 4.40977}	{2.55664, 6.39577}	{{1.63748, 3.58057}, {3., 7.91165}}	{84.2237, 58.1991}	inf	333.	429.558	{172.474, 467.204}	86.133	0.236725	newly	507.	558.46	{131., 543.}	110.398	0.425984	Reff	0.993103	0.650576	{0.212137, 0.928132}	53.8579	ar	5154.	18133.4	{16086., 25119.}	251.832
23	R0	{1.53657, 7.50352}	{2.27084, 7.03061}	{{0.608608, 0.444532}, {3., 11.0778}}	{81.5801, 65.1328}	inf	535.	688.007	{39.9486, 931.11}	144.172	0.633661	newly	479.	850.06	{17., 2194.}	182.881	0.748395	Reff	0.991395	0.556642	{0.0936217, 1.07149}	54.6187	ar	5928.	17449.4	{15527., 19917.}	194.356
24	R0	{1.71131, 6.24526}	{2.60276, 6.28926}	{{1.63633, 3.60833}, {3., 8.66669}}	{54.22, 40.394}	inf	964.	304.817	{214.069, 436.463}	73.4725	0.574106	newly	1016.	360.11	{121., 620.}	72.4793	0.617561	Reff	0.982634	0.600537	{0.128898, 0.923591}	52.1357	ar	6924.	17589.4	{15852., 23282.}	154.036
25	R0	{1.4412, 3.07729}	{2.37928, 5.08483}	{{1.35097, 2.0377}, {3., 9.15833}}	{67.4375, 101.607}	inf	606.	522.83	{127.381, 1276.58}	93.5693	0.470152	newly	1165.	914.73	{82., 4936.}	110.193	0.713289	Reff	0.935195	0.565238	{0.0936463, 1.32629}	55.4196	ar	5810.	18784.	{16975., 25122.}	223.304
26	R0	{1.4159, 4.93858}	{2.08022, 9.00614}	{{0.31955, 5.42253}, {3., 13.6816}}	{87.416, 82.3629}	inf	458.	344.351	{29.6458, 412.654}	65.9782	0.439777	newly	608.	249.22	{22., 299.}	78.7303	0.723106	Reff	0.962104	0.407579	{0.0672766, 0.674752}	59.2075	ar	5255.	17805.8	{16894., 17924.}	238.835
27	R0	{1.57465, 7.05414}	{2.45318, 5.51997}	{{1.38608, 2.1842}, {3., 9.46786}}	{60.0189, 50.8361}	inf	797.	300.722	{32.5388, 412.874}	89.3416	0.844902	newly	776.	411.12	{29., 459.}	102.149	0.854621	Reff	1.05486	0.514499	{0.0704026, 0.6296}	60.6096	ar	6359.	18292.3	{17066., 19605.}	187.659
28	R0	{1.41422, 4.62402}	{2.25824, 9.13907}	{{0.265963, 5.38909}, {3., 13.5302}}	{93.7849, 97.6435}	inf	570.	306.727	{23.3202, 537.576}	71.0308	0.621275	newly	804.	217.23	{13., 302.}	83.2301	0.927388	Reff	0.944699	0.471657	{0.060132, 0.668432}	50.6383	ar	5485.	17305.1	{16500., 17968.}	215.498
29	R0	{1.41901, 3.63017}	{2.24186, 4.62455}	{{1.66563, 3.54106}, {3., 6.87263}}	{66.2876, 29.772}	inf	600.	342.914	{156.07, 560.307}	88.7269	0.613608	newly	1020.	365.73	{106., 676.}	87.5598	0.816011	Reff	0.854528	0.351157	{0.0602372, 0.56119}	62.5988	ar	5979.	18221.9	{16903., 21390.}	205.989
30	R0	{1.60719, 4.72984}	{2.42903, 4.93288}	{{1.99744, 3.91781}, {3., 7.10667}}	{54.8886, 27.4854}	inf	850.	251.239	{16.2418, 260.76}	88.4322	0.920652	newly	1189.	305.11	{13., 727.}	82.6064	0.986509	Reff	0.978457	0.607283	{0.158522, 2.09273}	66.1213	ar	6479.	18859.5	{16885., 24659.}	191.087
31	R0	{1.38175, 6.26468}	{2.47774, 8.21571}	{{0.617702, 1.26937}, {3., 18.8576}}	{96.7563, 124.983}	inf	497.	310.689	{82.094, 319.944}	69.8514	0.440193	newly	506.	806.78	{67., 2967.}	175.542	0.619803	Reff	0.889156	0.560826	{0.0470767, 0.794679}	60.0232	ar	5397.	17763.6	{16463., 21598.}	229.138
32	R0	{1.4947, 8.8847}	{2.67937, 5.51724}	{{1.95935, 3.5176}, {3., 7.17188}}	{79.2582, 37.9018}	inf	437.	494.474	{78.4133, 770.044}	115.976	0.497064	newly	341.	563.21	{57., 1712.}	136.824	0.450518	Reff	0.948387	0.679953	{0.112233, 1.95215}	61.3331	ar	5407.	17512.9	{14967., 23464.}	223.893
33	R0	{1.55891, 8.28978}	{2.67422, 5.52802}	{{1.95687, 3.27608}, {3., 8.82888}}	{73.1055, 37.5378}	inf	655.	903.886	{147.208, 2382.72}	109.116	0.370794	newly	521.	861.25	{143., 2159.}	108.808	0.333414	Reff	0.96668	0.645298	{0.202419, 1.32822}	59.9775	ar	6239.	18650.6	{15908., 25450.}	198.935
34	R0	{1.60764, 10.4729}	{2.67265, 2.90937}	{{1.91565, 1.13532}, {3., 7.70088}}	{66.247, 72.22}	inf	772.	472.824	{145.114, 599.117}	103.857	0.727538	newly	469.	1118.15	{137., 3079.}	232.228	0.531541	Reff	0.879861	0.646248	{0.141635, 1.86835}	66.5596	ar	6495.	17457.	{15225., 24261.}	168.775
35	R0	{1.48527, 5.74485}	{2.50101, 8.30951}	{{2.03288, 1.1868}, {3., 18.7314}}	{83.3706, 136.134}	inf	650.	458.444	{30.1862, 915.634}	86.2455	0.577015	newly	769.	1187.28	{19., 3013.}	176.879	0.805777	Reff	0.956217	0.649218	{0.0674984, 1.60835}	59.1053	ar	5811.	18219.	{16433., 22400.}	213.526
36	R0	{1.61457, 9.93364}	{2.56422, 1.95501}	{{1.75773, 0.704608}, {3., 3.28579}}	{58.8177, 80.3193}	inf	799.	405.699	{137.622, 498.456}	68.2615	0.475609	newly	557.	688.72	{259., 1208.}	64.553	0.242036	Reff	1.02993	0.565069	{0.272437, 0.804501}	52.1436	ar	6338.	16416.5	{15554., 17420.}	159.018
37	R0	{1.47831, 7.08139}	{2.58699, 6.52039}	{{1.63898, 3.60163}, {3., 8.72226}}	{74.9963, 34.3378}	inf	564.	406.404	{208.139, 408.132}	75.8081	0.381718	newly	542.	518.84	{129., 540.}	98.2694	0.421906	Reff	0.993129	0.665956	{0.130408, 0.912581}	56.9344	ar	5530.	18207.8	{16268., 24900.}	229.256
38	R0	{1.73445, 9.8858}	{2.60775, 6.41415}	{{1.62427, 3.60521}, {3., 8.62118}}	{53.3827, 35.1176}	inf	986.	471.975	{191.722, 397.49}	87.4749	0.608878	newly	672.	628.61	{128., 556.}	109.246	0.510234	Reff	1.0313	0.65039	{0.0651078, 0.973733}	56.9152	ar	6842.	18229.4	{15949., 24926.}	166.433
39	R0	{1.66621, 11.4824}	{2.33518, 3.40361}	{{0.890721, 2.19469}, {3., 4.48099}}	{67.1168, 70.358}	inf	863.	326.145	{55.5055, 246.919}	91.8247	0.767104	newly	517.	415.06	{55., 367.}	92.646	0.496258	Reff	1.00256	0.419539	{0.161491, 0.7369}	60.4105	ar	6473.	16225.8	{15323., 16293.}	150.669
40	R0	{1.60694, 9.95741}	{2.30275, 12.336}	{{0.904944, 5.77826}, {3., 16.6492}}	{69.397, 40.6926}	inf	852.	438.476	{153.989, 578.651}	70.7326	0.502806	newly	560.	243.07	{69., 416.}	73.1054	0.601565	Reff	0.960147	0.566743	{0.136403, 1.17897}	52.8218	ar	6625.	16799.1	{15661., 18625.}	153.572
41	R0	{1.55128, 4.71911}	{2.43766, 4.73241}	{{1.96229, 3.82595}, {3., 7.12711}}	{62.029, 28.1387}	inf	710.	342.916	{136.044, 595.742}	91.0496	0.707069	newly	1008.	414.57	{90., 990.}	86.6319	0.806995	Reff	1.00632	0.520229	{0.192087, 0.844447}	64.6739	ar	6141.	18717.9	{16883., 23299.}	204.802
42	R0	{1.60063, 6.81118}	{2.63461, 7.06815}	{{2.27126, 0.126471}, {3., 19.0714}}	{74.4853, 74.5546}	inf	843.	899.174	{51.3484, 2086.93}	104.436	0.601963	newly	835.	997.04	{61., 2556.}	120.34	0.652414	Reff	0.933487	0.723494	{0.1239, 1.80128}	64.2023	ar	6528.	18361.5	{15467., 23069.}	181.272
43	R0	{1.56986, 5.44652}	{2.43007, 5.34074}	{{1.53032, 2.06618}, {3., 9.29464}}	{55.6389, 61.6845}	inf	706.	327.829	{17.3079, 358.671}	98.7521	0.919746	newly	846.	433.83	{11., 415.}	108.833	1.01584	Reff	0.905652	0.469416	{0.0754598, 0.597991}	61.2666	ar	6312.	18562.4	{17249., 19623.}	194.082
44	R0	{1.47523, 8.07785}	{2.55214, 1.93642}	{{1.75783, 0.5092}, {3., 3.28654}}	{72.9994, 76.0281}	inf	601.	565.223	{103.266, 522.187}	91.6162	0.453448	newly	507.	868.33	{171., 1727.}	115.288	0.305718	Reff	0.965243	0.557397	{0.323047, 0.886277}	50.4642	ar	5692.	16706.9	{15522., 19632.}	193.515
45	R0	{1.6417, 7.69429}	{2.60135, 6.3776}	{{1.60821, 3.6313}, {3., 8.71704}}	{59.4001, 31.4683}	inf	911.	431.824	{189.609, 488.295}	81.3323	0.568947	newly	777.	565.73	{126., 698.}	93.399	0.553773	Reff	0.893577	0.59379	{0.0902827, 0.945167}	53.3335	ar	6735.	17892.6	{15751., 24349.}	165.667
46	R0	{1.38865, 5.83473}	{2.25506, 6.95901}	{{0.646504, 0.650423}, {3., 9.29811}}	{93.2433, 67.2353}	inf	383.	685.73	{48.0831, 935.623}	178.527	0.56397	newly	442.	749.63	{32., 1200.}	185.505	0.724576	Reff	0.981081	0.518923	{0.0645398, 0.948136}	59.8411	ar	5233.	17587.	{15546., 20593.}	236.079
47	R0	{1.45289, 6.87903}	{2.55736, 6.46238}	{{1.64564, 3.59172}, {3., 8.54113}}	{76.0192, 34.0845}	inf	477.	426.249	{175.91, 565.855}	83.6827	0.370799	newly	487.	566.43	{119., 522.}	113.867	0.424124	Reff	1.01252	0.677193	{0.215852, 1.52861}	55.7027	ar	5379.	18181.	{16191., 24932.}	237.999
48	R0	{1.69416, 9.4831}	{2.53088, 7.84495}	{{1.80264, 3.2843}, {3., 10.8247}}	{49.3886, 34.8171}	inf	944.	970.894	{136.893, 956.868}	33.7534	0.194056	newly	702.	845.28	{85., 1308.}	71.7464	0.31332	Reff	1.03412	0.430901	{0.181418, 0.747162}	60.1973	ar	6925.	17957.4	{16109., 20895.}	159.312
49	R0	{1.47843, 7.16286}	{2.2752, 6.42026}	{{0.608732, 0.572506}, {3., 9.86255}}	{85.5234, 57.6684}	inf	577.	687.052	{53.1585, 1358.86}	140.352	0.637981	newly	548.	888.68	{44., 2060.}	175.85	0.691214	Reff	0.972659	0.536441	{0.0960543, 0.962967}	56.4097	ar	5449.	17551.2	{15575., 20634.}	222.099
50	R0	{1.68843, 5.72427}	{2.319, 5.41683}	{{1.65284, 0.380437}, {3., 14.6035}}	{39.0327, 50.6645}	inf	853.	932.75	{110.023, 2991.55}	109.323	0.542924	newly	913.	1482.56	{93., 5412.}	169.271	0.644495	Reff	0.853333	0.577236	{0.157741, 1.46784}	60.5589	ar	6800.	19196.6	{16322., 29415.}	183.54
